# Differential Proteomics of Large Extracellular Vesicles in Ovarian Cancer

**DOI:** 10.1002/pmic.70054

**Published:** 2025-10-02

**Authors:** Kazuhiro Suzuki, Yusuke Yamamoto, Masami Kitagawa, Eri Asano‐Inami, Kosuke Yoshida, Hiroaki Kajiyama, Akira Yokoi

**Affiliations:** ^1^ Department of Obstetrics and Gynecology Nagoya University Graduate School of Medicine Nagoya Aichi Japan; ^2^ Laboratory of Integrative Oncology National Cancer Center Research Institute Tokyo Japan; ^3^ Institute for Advanced Research Nagoya University Nagoya Aichi Japan; ^4^ Fusion Oriented Research for disruptive Science and Technology Japan Science and Technology Agency Kawaguchi Saitama Japan

**Keywords:** biomarker, extracellular vesicle, liquid biopsy, ovarian cancer, proteomics

## Abstract

Diverse extracellular vesicles (EVs) are present in all body fluids; however, knowledge of large EVs (lEVs) remains limited. Molecular EV profiles vary depending on EV size and the physiological circulatory system, even within the same patient. In this study, we aimed to characterize the proteomic profile of IEVs in ovarian cancer patients and identify lEV‐protein biomarkers. We collected tissue, serum, and ascites from patients with high‐grade serous ovarian cancer and concurrently separated small EVs (sEVs) and lEVs through sequential multistep centrifugation. Proteomic analysis of tissues and EVs revealed distinct EV profiles in serum and ascites, identifying 11 lEV‐specific proteins in serum and 14 in ascites that were absent in sEV. Of these, seven serum‐specific and 10 ascites‐specific proteins were further analyzed using transcriptomic databases, revealing candidate diagnostic and prognostic lEV‐protein biomarkers. Our findings underscore the importance of size‐based EV separation, as particle size influences biosynthetic mechanisms, in identifying lEV‐specific proteins with potential diagnostic and prognostic values.

**Summary:**

- This study underscores the importance of distinguishing extracellular vesicle (EV) subtypes and considering body fluid specificity in biomarker discovery.
- By isolating EVs based on size and stepwise separation and analyzing their proteomic profiles in ovarian cancer, we identified potential large EV (lEV)‐specific biomarkers that reflect disease pathology.
- These findings provide a foundation for lEV‐protein–based liquid biopsy approaches that could enhance the accuracy of early detection and patient stratification. Further validation in clinical settings may pave the way for more precise and personalized ovarian cancer diagnostics.

**Abbreviations**
AIM1: absent in melanoma 1
APOA5: apolipoprotein A5
APOC2: apolipoprotein C2
APOC3: apolipoprotein C3
APOC3: apolipoprotein C3
BLTP1: bridge‐like lipid transfer protein family member 1
C1RL: complement C1r subcomponent like
CFD: complement factor D
ECM1: extracellular matrix protein 1
EV: extracellular vesicles
F13B: coagulation factor XIII B chain
GEPIA: Gene Expression Profiling Interactive Analysis
IGHV3‐13: immunoglobulin heavy variable 3–13
IGHV3‐38: immunoglobulin heavy variable 3–38
IGKV1‐27: immunoglobulin kappa variable 1–27
IGLV8‐61: immunoglobulin lambda variable 8–61
lEV: large extracellular vesicles
LYZ: lysozyme
PCDHGC5: protocadherin gamma subfamily C, 5
PLEK1: pleckstrin‐1
PON3: paraoxonase 3
PTGDS: prostaglandin D2 synthase
RASL11A: RAS like family 11 member A
SERPINA10: serpin family A member 10
sEV: small extracellular vesicles
TRANK1: tetratricopeptide repeat and ankyrin repeat containing 1

## 1 Introduction

Extracellular vesicles (EVs) are heterogeneous, lipid bilayer‐enclosed particles released by cells and found in body fluids [1]. They contain diverse biomolecules, including proteins, nucleic acids, lipids, and metabolites, reflecting their cellular origin. The composition of EVs varies depending on the parent cell type and physiological state. Due to their ability to transport cellular information, EVs hold promise as biomarkers for various diseases [2, 3]. EVs are classified into small EVs (sEVs) and large EVs (lEVs) based on size. sEVs are defined as vesicles smaller than 200 nm in diameter, while lEVs are larger than 200 nm. For separation, sEVs are typically extracted using ultracentrifugation, size exclusion chromatography, or ultrafiltration. In contrast, lEVs are typically separated using lower‐speed centrifugation, density gradient centrifugation, or filtration [1]. Research on sEVs, previously known as exosomes, has been extensive, with studies focusing on their potential as biomarkers [4, 5, 6]. In contrast, research on lEVs as biomarkers is relatively scarce [7]. Furthermore, sEVs and lEVs require different separation methods, and the complexity of the extraction process may have led to incomplete separation in some studies analyzing lEVs. As a result, sEVs might have been inadvertently included in this analysis, potentially affecting the specificity of lEV research [8].

Ovarian cancer is a highly lethal gynecological malignancy with a poor prognosis, primarily due to late‐stage diagnosis and the lack of effective early screening methods [9, 10]. Therefore, the development of biomarkers for early detection and personalized treatment is essential. Previously, we identified an ovarian cancer‐specific sEV protein biomarker, analyzing sEVs separated from both serum and ascites [11]. While EVs have shown promise as a potential biomarker for ovarian cancer [12, 13, 14], a lEV‐specific biomarker that excludes sEV still remains unknown.

This study aimed to identify ovarian cancer lEV‐specific protein biomarkers unique to serum, ascites, or both. We collected ovarian cancer tissue, serum, and ascites fluid from the same patient, including proteins expressed in tumor tissue in our analysis. Furthermore, lEVs and sEVs were separately separated from serum and ascites using sequential multistep centrifugation, with lEVs collected as size‐separated fractions that may still contain small amounts of sEVs due to overlapping sedimentation properties. The gene information encoding the identified proteins was then integrated with public databases containing gene expression profiles and prognostic data. This approach enabled a comprehensive evaluation of the diagnostic and prognostic potential of these biomarkers in ovarian cancer.

## 2 Materials and Methods

### 2.1 Clinical Samples

Cancer tissue, serum, and ascites samples were collected from patients with stages III–IV high‐grade serous ovarian cancer during surgery at Nagoya University Hospital (Nagoya, Japan) between 2019 and 2021. A total of nine biological samples were obtained—three cancer tissues, three serum samples, and three ascites samples. From each serum and ascites sample, both sEVs and lEVs were separately separated, resulting in 15 total samples used for proteomic analysis. Written informed consent was obtained from all patients. The study was conducted with the approval (no. 2017‐0053) by the Institutional Review Board of Nagoya University Hospital. Cancer tissue sample were immediately frozen in liquid nitrogen and stored at −80°C until further use. Serum samples were centrifuged at 3000 rpm for 10 min at room temperature. Ascites samples were centrifuged at 300 × *g* undiluted for 5 min at 4°C to remove cell debris. The serum and ascites samples were stored at −80°C until further use.

### 2.2 Separation of EVs From Serum and Ascites Samples

Approximately 1 mL of each sample (serum or ascites) was centrifuged at 10,000 × *g* for 40 min at 4°C in the Kubota Model 7000 ultracentrifuge. The pellet was washed in PBS and centrifuged again under the same conditions to suspend the pellet and extract lEVs. The supernatant was filtered using a 0.22‐µm filter (Millex‐GV Syringe Filter Unit, Millipore). Ultracentrifuged at 55,000 rpm for 120 min at 4°C using MAX‐XP (Beckman Coulter Inc., USA). The pellet was washed with PBS, ultracentrifuged with the same conditions, and resuspended in PBS to extract sEVs [15].

### 2.3 Protein Quantification and Size Analysis of EVs

Protein concentration of EVs and cell lysates was quantified using a Qubit protein assay kit (Thermo Fisher Scientific) with the Qubit 4.0 Fluorometer (Invitrogen Co., MA, USA), according to the manufacturers’ protocol. The size distribution and particle concentration in the EV preparations were analyzed using a NanoSight NS300 (Malvern Panalytical Ltd., UK) nanoparticle tracking analyzer. Samples were diluted in PBS, injected into the measuring chamber, and EV flow was recorded in triplicate measurements (30 s each) at room temperature. Equipment settings for data acquisition were kept constant between measurements, with camera level set to 13.

### 2.4 Cryo‐Transmission Electron Microscopic Imaging

The morphology of sEVs and lEVs were visualized using a cryo‐transmission electron microscope (cryo‐TEM) (Terabase Inc., Okazaki, Japan) that could generate high‐contrast images of the nanostructures of biological materials without requiring staining procedures that could damage the samples. The natural structure of the sample distributed in the solution was observed by preparing the sample using a rapid vitreous ice‐embedding method. For cryo‐electron microscopic experiments, 2.5 uL of each sample solution was applied to standard Copper Quantifoil grids R1.2/1.3 (Quantifoil Micro Tools GmbH, Germany) and vitrified by rapid plunging into liquefied ethane using a Vitrobot Mark IV (Thermo Fisher Scientific) at 95% humidity and 4°C. The frozen grid was then mounted on a cryo‐transfer specimen holder (Model626, Gatan, CA, USA) at liquid nitrogen temperature and loaded into a JEM‐2200FS microscope (JEOL, Tokyo, Japan), equipped with a field emission gun and an omega‐type energy filter, operated at 200 kV. A slit width of 15 eV was used to obtain a zero‐energy‐loss electron beam. Images were recorded using a DE‐20 direct electron detection device (Direct Electron LP, CA, USA) at 30,000× magnification, corresponding to a pixel size of 1.96 Å on the specimen.

### 2.5 Mass Spectrometry

To obtain tissue proteins, small specimens of ovarian tissue were shredded and crushed using a gentleMACS Dissociator in protein mode with 7 M urea. The supernatant was collected after centrifugation at 15,000 rpm for 15 min at 4°C. For EV proteins, the samples were processed according to the Easy Pep protocol. Both the tissue and EV protein samples were then prepared for mass spectrometry (MS). The proteins were reduced, alkylated, and digested with trypsin at 37°C. The peptides were analyzed by LC‐MS using an Orbitrap Fusion mass spectrometer coupled to an UltiMate 3000 RSLCnano LC system with a nano HPLC capillary column (150 mm × 75 µm i.d.). A linear gradient was used for reversed‐phase chromatography (5%–40% B for 100 min), with solvent A (2% acetonitrile and 0.1% formic acid) and solvent B (95% acetonitrile and 0.1% formic acid) at an estimated flow rate of 300 nL/min. Precursor ion scans were performed with a mass‐to‐charge ratio (m/z) of 400–1600 before MS/MS analysis. For MS/MS, higher‐energy collisional dissociation (HCD) fragmentation was used with a normalized collision energy of 35% and an isolation width of 1.6 m/z. Only precursors with charge states of 2–6 were sampled for MS2, and the dynamic exclusion duration was set to 15 s with a 10 ppm tolerance. The instrument was operated in top‐speed mode with 3 s cycles. Reversed‐phase chromatography was performed with a linear gradient (0 min, 5% B; 100 min, 40% B) of solvent A (2% acetonitrile with 0.1% formic acid) and solvent B (95% acetonitrile with 0.1% formic acid) at an estimated flow rate of 300 nL/min. A precursor ion scan was carried out using a 400–1600 mass to charge ratio (m/z) prior to MS/MS analysis. Tandem MS was performed by isolation width of 1.6 m/z with the quadrupole, HCD fragmentation with normalized collision energy of 35%, and rapid scan MS analysis in the ion trap. Only those precursors with charge state 2–6 were sampled for MS/MS analysis. The dynamic exclusion duration was set to 15 s with a 10 ppm tolerance. The instrument was run in top speed mode with 3 s cycles.

### 2.6 Proteomic Data Analysis

The raw data was processed using either Proteome Discoverer 2.4 (Thermo Fisher Scientific) in conjunction with MASCOT search engine, version 3.0.0 (Matrix Science Inc., Boston, MA, USA) for protein identification. Peptides and proteins were identified against human protein database in UniProt (release 2024_05), with a precursor mass tolerance of 10 ppm, a fragment ion mass tolerance of 0.8 Da. Fixed modification was set to carbamidomethylation of cysteine, and variable modification was set to oxidation of methionine. Two missed cleavages by trypsin were allowed.

### 2.7 Western Blotting Analysis

The samples of EVs prepared with adjusted amounts of protein were loaded onto polyacrylamide gels for electrophoretic separation of proteins at 30 mA. After blocking with Blocking One (Nacalai Tesque Inc., Japan) for 1 h at room temperature, the membranes were incubated overnight at 4°C with the following primary antibodies: mouse monoclonal anti‐CD9 (CBL162; Merck; 1:100), rabbit monoclonal anti‐CD63 (EXOAB‐CD63A‐1; System Biosciences, LLC, CA, USA; 1:1000), mouse monoclonal anti‐CD81 (sc‐166029; Santa Cruz Biotechnology, TX, USA; 1:100), mouse monoclonal anti‐ApoA1 (#3350; Cell Signaling Technology, MA, USA; 1:1000), mouse monoclonal anti‐ApoB‐100 (C1.4) (sc‐13538; Santa Cruz Biotechnology, TX, USA; 1:1000), rabbit polyclonal anti‐Calnexin (ab22595; Abcam, Cambridge, UK;1:500), and mouse monoclonal anti‐GM130 (Cat# 610822; BD Biosciences, CA, USA; dilution 1:500). The membranes were subsequently washed three times for 5 min each using TBS with 0.1% Tween 20 and incubated for 1–3 h at room temperature with secondary HRP‐conjugated mouse anti‐rabbit IgG (NA934‐1ML; Cytiva Lifesciences, USA; 1:5000) or anti‐mouse IgG (NA931‐1ML; Cytiva; dilution) antibodies. The protein ladder is MagicMarkTM XP Western Protein Standard (Thermo Fisher Scientific). The membranes were imaged using ImageQuant LAS 4010 (GE Healthcare, IL, USA). The uncropped blots are shown in Supporting information.

### 2.8 Statistical Analysis

The unpaired Student's *t*‐test was used to compare means between two groups. Kaplan–Meier analysis with log‐rank test and Cox regression analysis were performed using IBM SPSS Statistics version 30 (IBM Japan, Japan). The other analyses were conducted using R version 4.4.2 (R Foundation for Statistical Computing, http://R‐project.org). Statistical significance for all analyses was defined as *p* < 0.05.

## 3 Results

### 3.1 EVs Appearances of Patients With Ovarian Cancer

**FIGURE 1:**
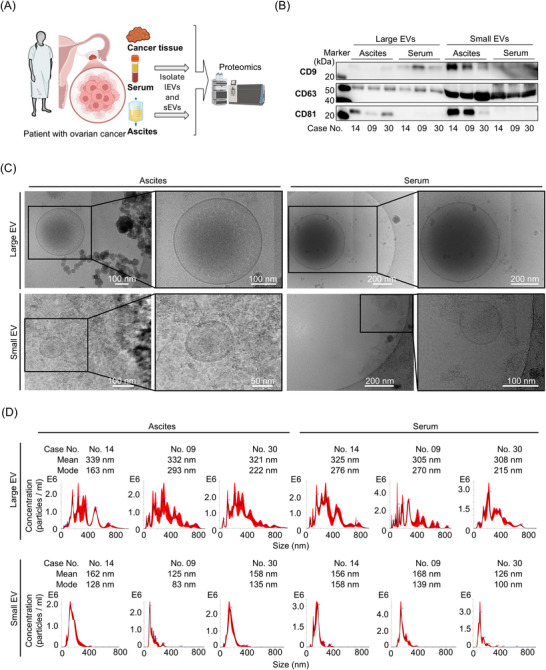
Characterization of EVs. (A) Schematics of sample preparation for proteomics. Cancer tissue, serum, and ascites were all obtained from the same patient. Small EVs (sEVs) and large EVs (lEVs) were extracted concurrently from patient serum and ascites using sequential multistep centrifugation, as described in Section 2. (B) Protein expression levels of EV markers (CD9, CD63, and CD81) were analyzed in each EV sample. (C) Representative images showing the morphology of EVs were obtained using cryo‐transmission electron microscopy. (D) Size distribution and concentration were analyzed by nanoparticle tracking assays in each EV sample.

We collected fresh‐frozen cancer tissue, serum, and ascites from the same patients with high‐grade serous ovarian cancer (Figure 1A). sEVs and lEVs were separated from serum and ascites using sequential multistep centrifugation. Proteomic analysis was performed on 15 samples from three cases. As detailed in Table S1, cases 14 and 30 underwent primary debulking surgery, while case 09 received neoadjuvant chemotherapy followed by interval debulking surgery. To characterize EVs, nanoparticle tracking analysis, cryo‐EM, and Western blotting were performed to assess particle size, morphology, and marker protein expression, respectively (Figures 1B–D and S1).

### 3.2 Characteristics of the EV‐Protein Profile in Ovarian Cancer

**FIGURE 2:**
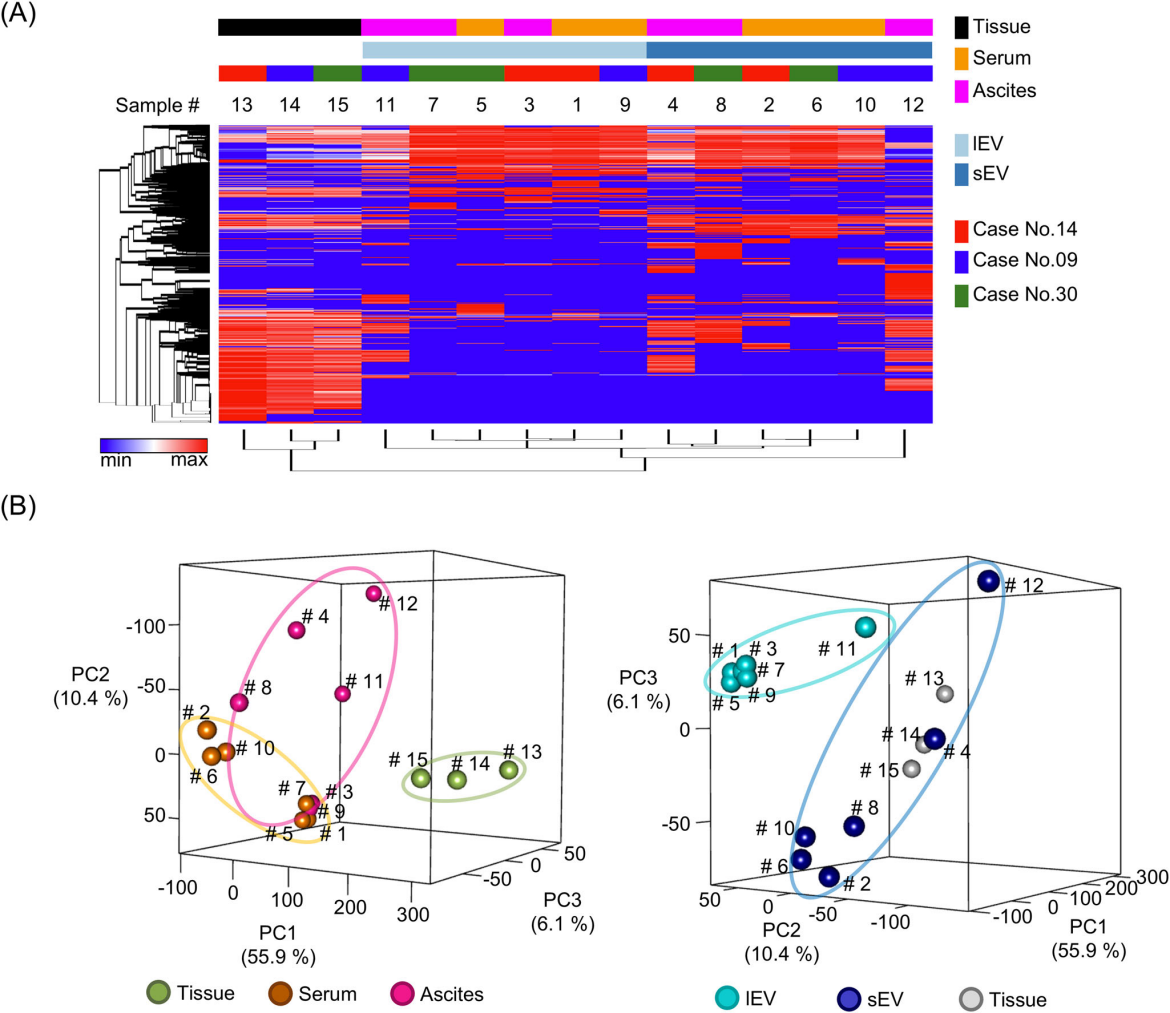
Characteristics of the EV‐protein profile in ovarian cancer. (A) Hierarchical clustering heatmap of 831 protein expressions across 15 samples including cancer tissue, serum, and ascites, based on mass spectrometry analysis. (B) Principal component analysis of 15 samples using mass spectrometry data. The left side of the figure indicates the color codes for the collected samples, while the right side represents the color code for separated lEV and sEV. lEV, large extracellular vesicles; Pt, patient; sEV, small extracellular vesicles.

First, to confirm the differences in protein expression between lEVs and sEVs, we analyzed their individual protein expression profiles. To identify proteins not only detectable in circulation but also directly associated with tumor biology, we first selected candidate biomarkers based on 831 proteins that were commonly expressed across all three ovarian cancer tissue samples. Hierarchical clustering analysis showed distinct clustering patterns among tissues. sEVs and lEVs (Figure 2A). PCA showed a consistent trend in protein expression profiles across tissue, serum, and ascites fluid, respectively (Figure 2B). Additionally, PCA using PC2 as the main axis revealed different expression patterns between lEV and sEV.

### 3.3 Identification of Unique Serum lEV‐Protein Profile in Ovarian Cancer

**FIGURE 3:**
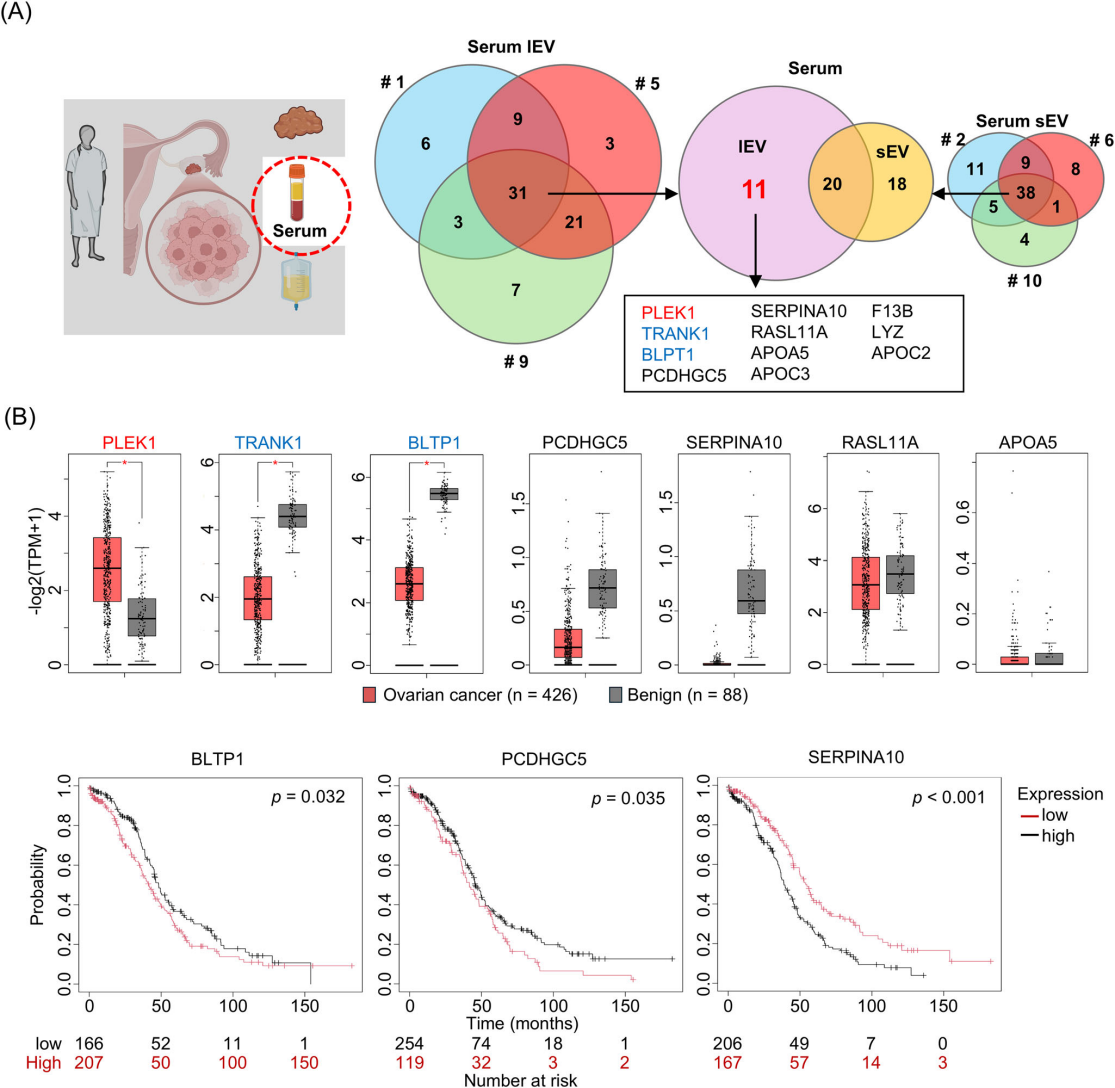
Identification of unique serum lEV‐protein profile in ovarian cancer. (A) lEVs and sEVs were separated from serum samples collected from three patients. Venn diagrams display the proteins in identified serum lEVs and sEVs, represented by their gene symbols. (B) The gene expression profiles of ovarian cancer tissues were analyzed for the genes for seven target protein genes using the GEPIA database. Box/dot plots illustrate the differences in gene expression levels between ovarian cancer and normal tissue. Kaplan–Meier curves of the genes of the three target proteins with significant differences obtained by Kaplan–Meier Plotter. FC, fold change; GEPIA, Gene Expression Profiling Interactive Analysis; lEV, large extracellular vesicles; sEV, small extracellular vesicles.

The primary objective of this study was to identify ovarian cancer‐specific proteins in lEVs. To achieve this, we focused solely on serum samples to pinpoint proteins specific to lEVs in ovarian cancer (Figure 3A). A total of 31 proteins were commonly detected across the three lEV samples, while 38 proteins were shared among the three sEV samples. Of these, 11 were identified as lEV‐specific proteins: PLEK1, TRANK1, BLTP1, PCDHGC5, SERPINA10, RASL11A, APOA5, APOC3, F13B, LYZ, and APOC2. Among these 11 proteins, seven were exclusive to serum: PLEK1, TRANK1, BLTP1, PCDHGC5, SERPINA10, RASL11A, and APOA5. To assess the relative changes in gene expression levels associated with ovarian cancer, we analyzed the mRNA profiles of 426 ovarian cancer tissues and 88 normal tissues using the Gene Expression Profiling Interactive Analysis (GEPIA) database (http://gepia.cancer‐pku.cn/), which is based on The Cancer Genome Atlas (TCGA) and Genotype‐Tissue Expression (GTEx) data. The results revealed significantly higher gene expressions of PLEK1 in ovarian cancer tissue compared to normal ovarian tissues (Figure 3B). In contrast, TRANK1 and BLTP1 exhibited significantly lower gene expression in ovarian cancer tissue than in normal ovarian tissue. To explore the association between gene expression and prognosis, we employed the Kaplan–Meier Plotter (KM plotter) (https://kmplot.com/), another open‐access database based on TCGA and GTEx data. Analysis of the seven targets indicated a correlation between gene expression and prognosis for three proteins: BLTP1, PCDHGC5, and SERPINA10.

### 3.4 Identification of Unique Ascites lEV‐Protein Profile in Ovarian Cancer

**FIGURE 4:**
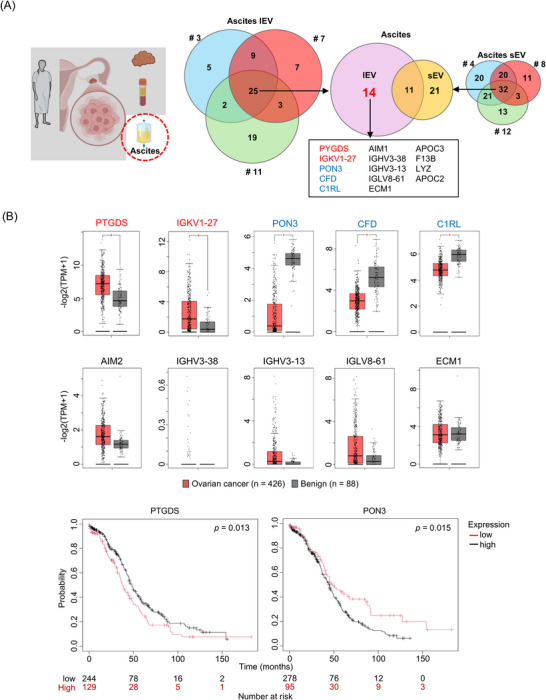
Identification of unique ascites lEV‐protein profile in ovarian cancer. (A) From ascites samples collected from three patients, lEV and sEV were separated. Venn diagrams of proteins in ascites lEVs and sEVs. Proteins are identified by their gene symbols. (B) The gene expression profiles of ovarian cancer tissues were examined for the genes of the 10 target proteins using the GEPIA database. Box/dot plots depict the differences in gene expression levels between ovarian cancer and normal tissue. Kaplan–Meier curves of the genes of the two target proteins with substantial differences were collected using the Kaplan–Meier Plotter. FC, fold change; GEPIA, Gene Expression Profiling Interactive Analysis; lEV, large extracellular vesicles; sEV, small extracellular vesicles.

Next, we focused exclusively on ascite samples to identify lEV‐specific proteins in ovarian cancer (Figure 4A). A total of 25 proteins were commonly detected across the three lEV samples, while 32 proteins were shared among the three sEV samples. Of these, 14 were identified as lEV‐specific proteins: PTGDS, IGKV1‐27, PON3, CFD, C1RL, AIM1, IGHV3‐38, IGHV3‐13, IGLV8‐61, ECM1, APOC3, F13B, LYZ, and APOC2. Among these 14 proteins, 10 were exclusive to serum: PTGDS, IGKV1‐27, PON3, CFD, C1RL, AIM1, IGHV3‐38, IGHV3‐13, IGLV8‐61, and ECM1. To assess their mRNA expression profiles, using the GEPIA database. The results revealed significantly higher gene expression in ovarian cancer tissue compared to normal ovarian tissues for two of the 10 targets, including PTGDS and IGKV1‐27 (Figure 4B). In contrast, PON3, CFD, and C1RL exhibited significantly lower gene expression in ovarian cancer tissue than in normal ovarian tissue. To investigate the association between gene expression and prognosis, we analyzed the 10 targets using the KM plotter. The results indicated a correlation between gene expression and prognosis for two proteins: PTGDS, and PON3.

### 3.5 Identification of Universal lEV‐Protein Profile in Ovarian Cancer

**FIGURE 5:**
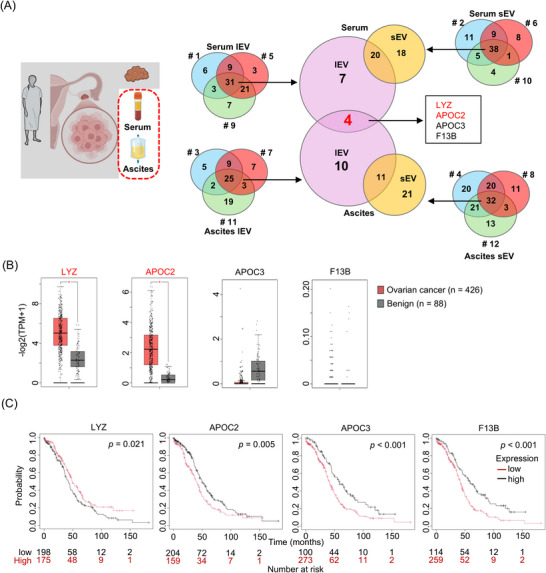
Identification of universal ascites and serum lEV‐protein profile in ovarian cancer. (A) From ascites and serum samples obtained from three patients, lEV and sEV were separated. Venn diagrams for proteins in lEVs and sEVs. Proteins are identified by their gene symbols. (B) The gene expression profiles of ovarian cancer tissues were analyzed for the genes of the four target proteins using the GEPIA database. Box/dot plots depict the difference in gene expression levels between ovarian cancer and normal tissue. Kaplan‐Meier curves of the genes of the four target proteins with significant differences obtained by Kaplan–Meier Plotter. FC, fold change; GEPIA, Gene Expression Profiling Interactive Analysis; lEV, large extracellular vesicles; sEV, small extracellular vesicles.

Finally, we focused on both serum and ascites samples to identify proteins specific to lEV in ovarian cancer (Figure 5A). All lEV serum and ascites samples had four proteins in common: LYZ, APOC2, APOC3, and F13B. We investigated the mRNA expression profiles by using GEPIA, which revealed significantly higher gene expressions in ovarian cancer tissue than in normal ovarian tissues for two of the four targets, LYZ and APOC2 (Figure 5B). We then analyzed the gene expression and prognosis of four targets using a KM plotter. The results indicated a correlation between gene expression and prognosis for all four targets.

## 4 Discussion

This study uniquely identifies lEV‐specific protein biomarkers of ovarian cancer through a detailed analysis of EVs separated from both serum and ascites fluid. Sequential multistep centrifugation separated lEV and sEV from the same sample, enabling the identification of lEV‐specific markers. In particular, tumor tissue, serum, and ascites were obtained from the same patient, allowing targeted analysis of tumor‐expressed proteins for more reliable biomarker identification. This comprehensive approach distinguishes serum‐specific, ascites‐specific, and shared lEV biomarkers, offering new insights into ovarian cancer diagnosis and prognosis.

lEVs, which are easier to separate than sEVs, are emerging as promising candidates for cancer research, with ongoing studies exploring their functions and biomarker potential relative to sEVs [16, 17]. Although lEVs are detected more frequently than circulating tumor cells in cancer patients, further research is required to clarify their cancer‐specific roles and therapeutic potential [3, 18]. lEV biosynthesis differs from that of sEV, as lEVs bud directly from the plasma membrane whereas sEVs originate from intracellular vesicle trafficking [19]. As a result, even when secreted by the same cell, their contents and properties are heterogeneous and should be analyzed separately. The sequential multistep centrifugation method enables precise size‐based separation, preserving EV integrity and distinct molecular compositions [15]. This method enhances the accuracy of comparative analysis, facilitating a more accurate comparative analysis of the unique biological functions of lEV and sEV populations (Figure S2).

The EVs used in this investigation were extracted from samples of ascites fluid and serum, respectively. EVs are released into the bloodstream by cancer cells and can travel throughout the body after entering circulation. However, ovarian cancer is the exclusive cause of ascites, because EVs are released straight from cancer cells into the abdominal cavity's ascites. Significant expression changes between normal and ovarian cancer samples were seen for a few of the potential proteins. Based on these findings, they appear as possible ovarian cancer diagnostic indicators. In particular, it was discovered that PTGDS, IGKV1‐27, PON3, CFD, and C1RL were specific to ascites‐IEVs, whereas PLEK1, YRANK1, and BLTP1 were specific to serum lEVs. Furthermore, ovarian cancer prognoses for several candidate proteins differed significantly from those of corresponding gene expression profiles. These proteins can be used as prognostic biomarkers for ovarian cancer because of these findings. Specifically, it was discovered that PTGDS and PON3 were specific to ascites‐IEV, whereas BLTP1, PCDHGC5, and SERPINA10 were specific to serum lEVs. Non‐circulating IEVs or proteins not involved in the EV secretion mechanism may be biomarkers shared by ascites and serum. However, in the current study, a thorough hierarchical analysis of lEV‐protein biomarkers in bodily fluid is valuable.

This study has significant drawbacks. First, we did not conduct a functional analysis of the identified potential proteins. The function of the lEV protein is unclear and should be investigated further. Functional investigation could show how these proteins contribute to the pathogenesis of ovarian cancer and help discover possible treatment targets. The second limitation is the limited subjectivity of the analysis, as it only covered high‐grade serous ovarian cancer and did not examine other histologic subtypes. Ovarian cancer is recognized to have many histologic subtypes, each with a distinct molecular profile and clinical course [20]. A thorough analysis incorporating subtypes other than high‐grade serous, such as clear cell carcinoma and endometrial carcinoma, could have provided a better insight into the EV profile of ovarian cancer and found subtype‐specific biomarkers. Third, the study's small patient sample size is a limitation. Future analysis of a larger sample of patients would have allowed a more precise assessment of the lEV‐protein profile. This would validate the candidate biomarker's sensitivity and specificity, allowing for a more reliable assessment of their clinical application potential. Fourth, differential ultracentrifugation may co‐separate non‐EV particles such as lipoproteins and protein aggregates with similar density and size. This limitation has been highlighted in the MISEV2023 guidelines, and we acknowledge that our EV fractions may contain non‐vesicular contaminants (Figure S3). Therefore, our comparative proteomic analysis reflects separated EV populations rather than strictly purified subtypes. Future analysis or additional purification steps may help improve the specificity of EV subtype characterization.

This study successfully identified diagnostic and prognostic indicators for ovarian cancer patients by analyzing EVs in serum and ascites. A hierarchical analysis, that took into account differences in EV secretion mechanisms (sEV and lEV) and circulation (hematogenous and exudative) resulted from the development of more specific lEV‐protein biomarkers. This comprehensive strategy is a significant step toward achieving early detection and individualized treatment for ovarian cancer, and it is expected to be used clinically in the future.

## Author Contributions

Conceptualization: Kazuhiro Suzuki and Akira Yokoi. Methodology: Kazuhiro Suzuki, Akira Yokoi, Kosuke Yoshida, Masami Kitagawa, Eri Asano‐Inami, and Yusuke Yamamoto. Investigation: Kazuhiro Suzuki, Masami Kitagawa, and Eri Asano‐Inami. Supervision: Akira Yokoi and Hiroaki Kajiyama. Writing – original draft: Kazuhiro Suzuki and Akira Yokoi. Writing – review and editing: All authors.

## Conflicts of Interest

The authors declare no conflicts of interest.

## Supporting information




**Supporting File**: pmic70054‐sup‐0001‐SuppMat.docx.

## Data Availability

The data supporting the results of this study are openly available in the ProteomeXchange Consortium via the PRIDE partner repository, reference number PXD056996.
