# Supplementary material for: Differential Proteomics of Large Extracellular Vesicles in Ovarian Cancer
Source: Proteomics. 2025 Oct 2;26(2-3):95–104. doi: 10.1002/pmic.70054 (PMC12976830; doi:10.1002/pmic.70054)
Supplement: Supplementary file 1 — Supporting File: pmic70054‐sup‐0001‐SuppMat.docx. [file PMIC-26--s001.docx]

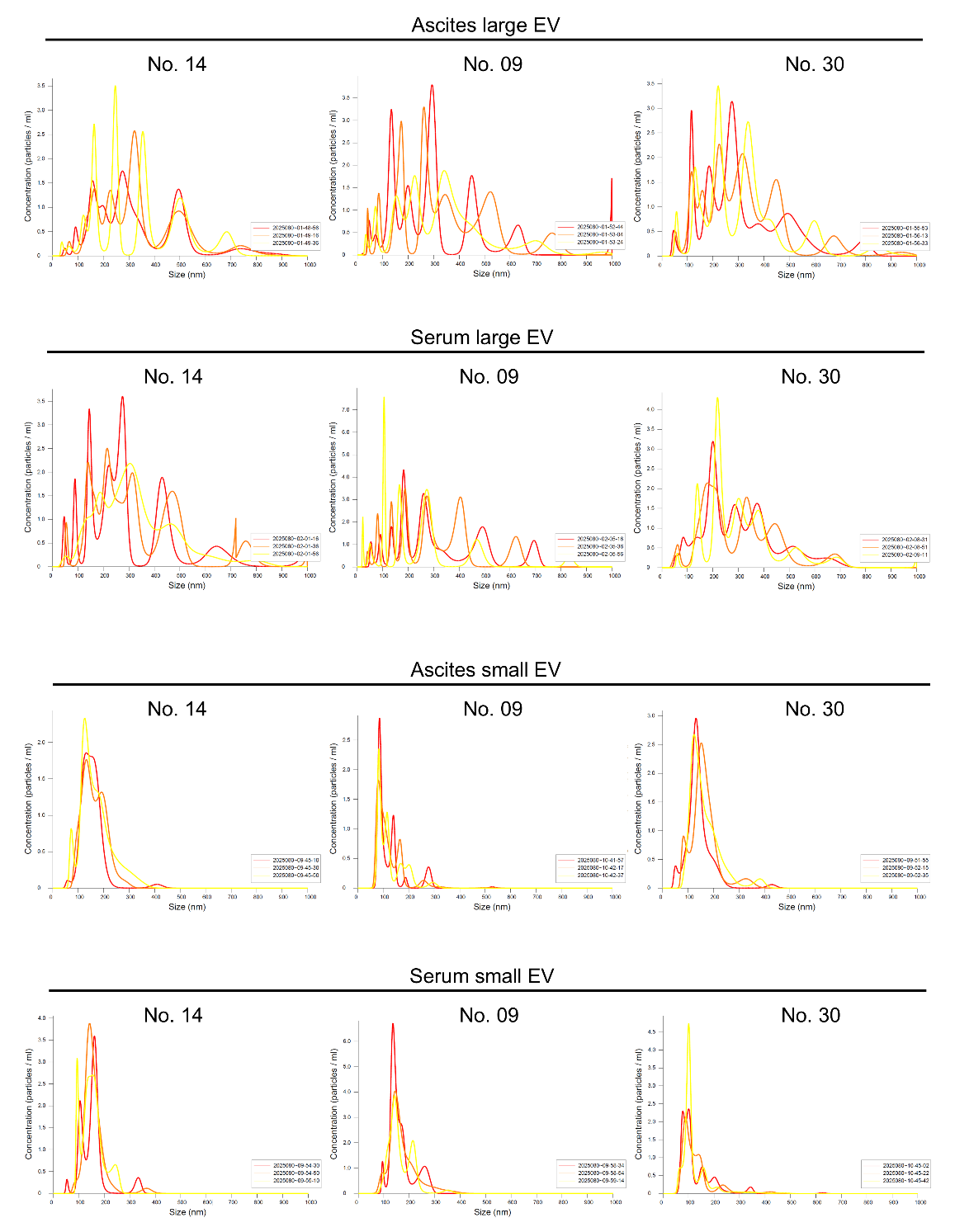


**Supplementary Figure 1**

Size distribution and concentration were analyzed by nanoparticle tracking assays in each EV sample. The number represents the case ID.


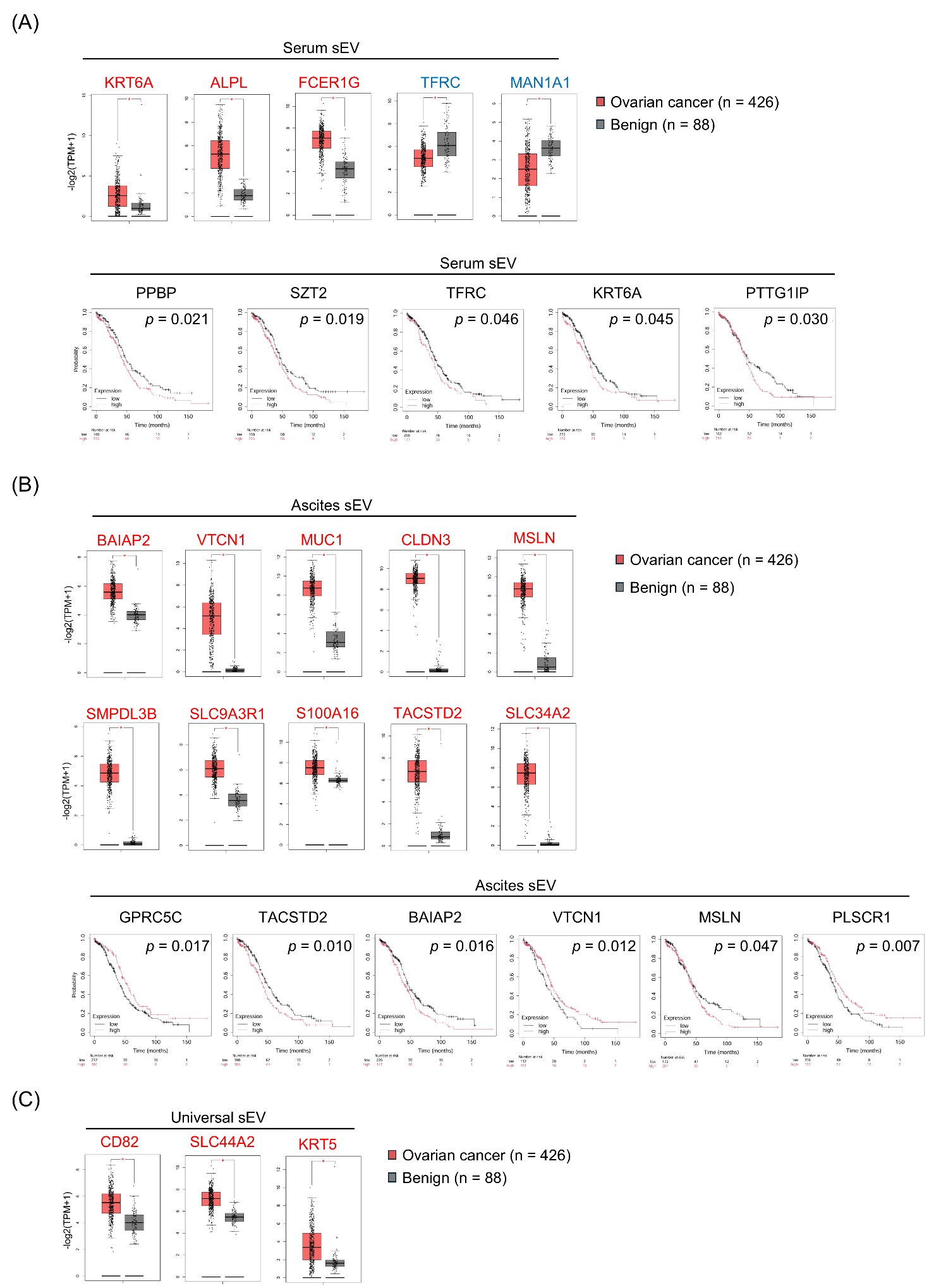
**Supplementary Figure 2**

Identification of unique sEV-protein profile in ovarian cancer. The gene expression profiles of ovarian cancer tissues were analyzed for the genes of the target proteins identified in (Figure 3A and Figure 4A) by referencing the GEPIA database. Box/dot plots show the difference in gene expression levels between ovarian cancer and normal tissue. Kaplan-Meier curves of the genes of the target proteins with significant differences obtained by Kaplan-Meier Plotter. (A) unique serum sEV-protein profile, (B) unique ascites sEV-protein profile, and (C) universal serum and ascites sEV-protein profile. lEV, large extracellular vesicles; sEV, small extracellular vesicles; GEPIA, Gene Expression Profiling Interactive Analysis; FC, fold change.


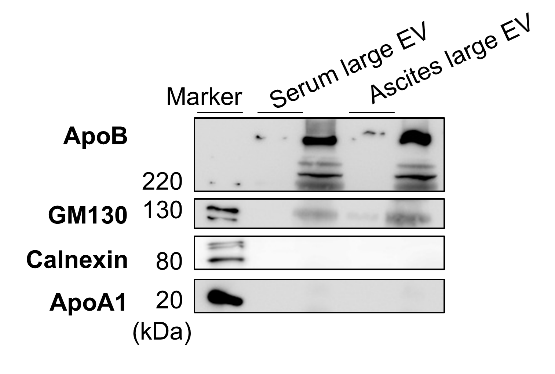


**Supplementary Figure 3**

Protein expression levels of non-EV markers (ApoA1, ApoB, Calnexin, and GM130) for representative lEV samples

| **Case ID** |  | 14 | 09 | 30 |
| --- | --- | --- | --- | --- |
| **Sample No (#)** |  | 1.2.3.4.13 | 5.6.7.8.14 | 9.10.11.12.15 |
| **Age** |  | 71 | 38 | 71 |
| **FIGO Stage** |  | IIIC | IVB | IIIC |
| **BRCA1/2 mutation** |  | n/a | negative | negative |
| **Histology** |  | Serous | Serous | Serous |
| **Sample collect** |  | PDS | IDS | PDS |
| **PFS (months)** |  | 65 | 17 | 18 |
| **OS (months)** |  | 65 | 41 | 43 |

**Supplementary Table 1 Patient characteristics.**

Abbreviations: BRCA, breast cancer susceptibility; PFS, progression-free survival; OS, overall survival; PDS, primary debulking surgery; IDS, interval debulking surgery.


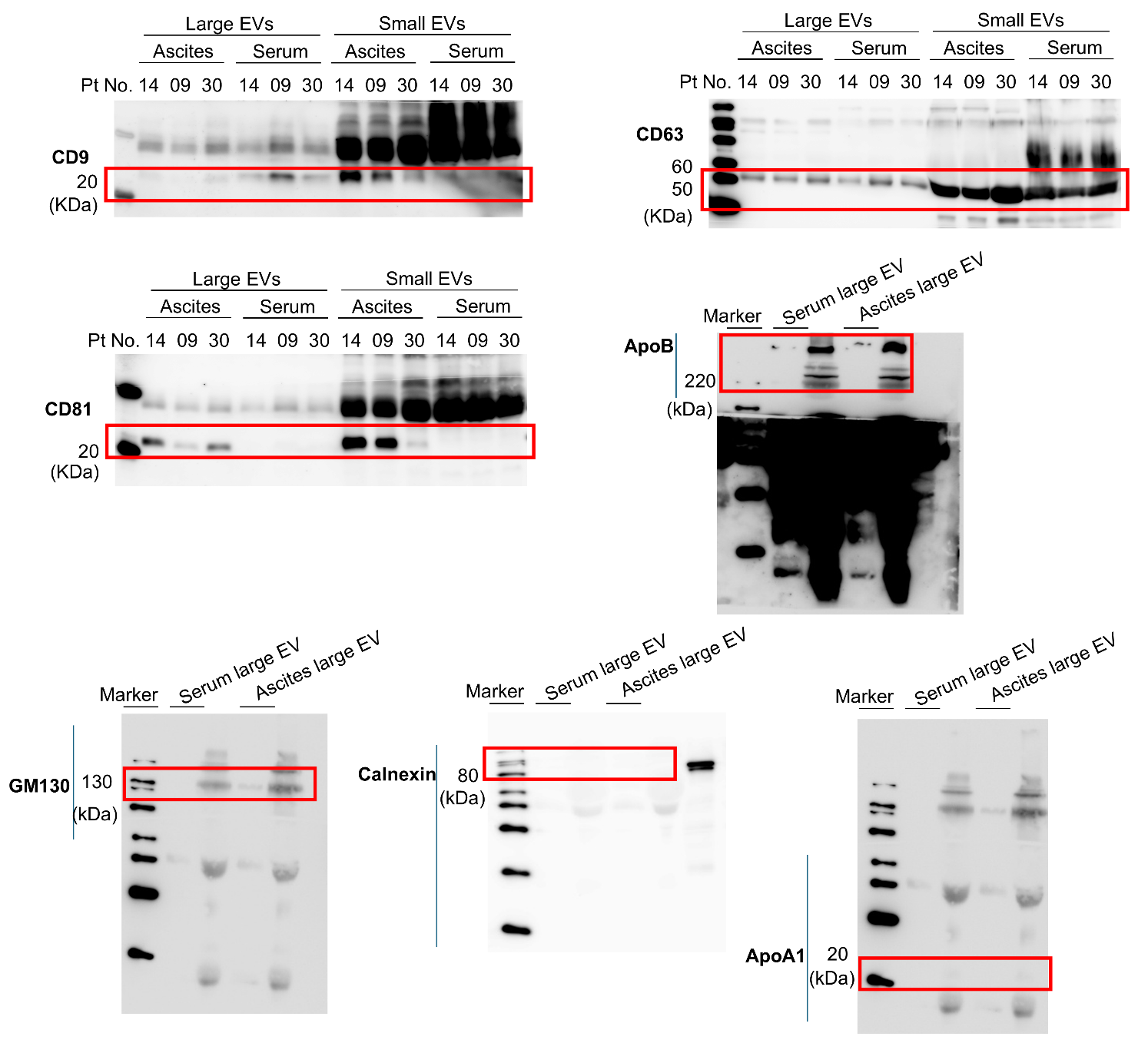


Full unedited gel for Figures and Supplementary Figures.
